# Quantum Fisher information of two atoms with dipole–dipole interaction under the environment of phase noise lasers

**DOI:** 10.1038/s41598-021-99449-9

**Published:** 2021-10-27

**Authors:** Yu Chen, Zheng-wen Long, Zhi He, Shen-tong Ji

**Affiliations:** 1 School of Physics and Electronic Science, Guizhou Education University, Guiyang, 550018 China; 2 College of Physics, Guizhou University, Guiyang, 550025 China; 3 College of Mathematics and Physics Science, Hunan University of Arts and Science, Changde, 415000 China

**Keywords:** Quantum information, Quantum optics

## Abstract

We investigate the parameter estimation problems of two-atom system driven by the phase noise lasers (PNLs) environment. And we give a general method of numeric solution to handle the problems of atom system under the PNLs environment. The calculation results of this method on Quantum Fisher Information (QFI) are consistent with our former results. Moreover, we consider the dipole–dipole (*d*–*d*) interaction between the atoms under PNLs environment with the collective decay, and the results show that larger *d*–*d* interaction and smaller collective decay rate lead to larger QFI of the two-atom system. So the collective decay will destroy the QFI while the *d*–*d* interaction will preserve the QFI, these results can be used to protect the QFI of two-atom system driven by the PNLs environment.

## Introduction

Parameter estimation is very important in different scientific field^1–3^, and QFI is a quantity which indicates the sensitivity of a parameter^4–7^. The estimated parameter usually refers to system frequency, the phase difference during the evolution, field strength and so on. How to increase the precision of estimation is the essential question on parameter estimation or metrology^8–15^. These years, QFI has many important applications, such as frequency standard in quantum technology^16^, the measurement of gravitational acceleration^17^, clock synchronization^18^, atomic clock^19^, et.al.

PNL is one kind of noise model which represent the inevitable phase noise in laser in the real world. In 1976, G.S.Agarwal provided the phase diffusion model of the PNL^20^, and he also discussed the analytical solution of one atom under the driven of PNL and in considering the spontaneous emission. Although he gave us the spectrum, the master equation still includes a random variable, which make us hard to use for some physical problems. After that, many researchers, such as J.H.Eberly, have investigated the PNL model^21–27^. They mainly consider the laser instability on the phase, and this instability can be regarded as a phase noise^20,21,28,29^. In 2010, based on Agarwal’s PNLs model^20,21,25,30^, and by using the Zwanzig–Nakajima projection operator technique, J.D.Cresser derived a master equation without a stochastic variable^31^. After that, there are some researchers studied the physical problems on PNLs with this J.D.Cresser’s master equation, such as QFI^1,32^, the discord of two-qubit *X* state^33^, and the discord and the entanglement of three-qubit GHZ state and *W* state^34^. Although they investigated multi-atom PNLs problems by using this master equation, their calculations are with no interactions between atoms and no spontaneous emissions, and do not focus on more complicated problems with *d*–*d* interactions and collective decay (or cooperative emission) of multiple atoms when the atoms are much closer to each other. Because there are two or multiple random noises inside their master equations and it is difficult to handle these complicated problems by mathematics. Generally, these problems can be solved only by numeric calculations.

In this paper we try to handle these problems. In our former research^1^, we have investigated the analytical results of QFI of GHZ state that atoms are separated far from each other, no interactions with them. On the base of above researchers’, we follow the way of derivation of J.D.Cresser’s master equation^31^ to derive the master equation and discuss the non-Markovian characters^35,36^ of QFI of two-qubit driven by the PNLs, which include two independent phase noise. In detail, we firstly try to study the QFI of maximal entanglement state of two atoms without the *d*–*d* interaction. We find that QFI decays slowly with oscillating when the ratio of diffusion rate and coupling coefficient is small, and this corresponds to non-Markovian region^37,38^. And when the ratio increases, QFI will decay fast without oscillating, which corresponds to Markovian region. Our numeric calculations are consistent with our former analytical calculations by comparison. Secondly, we consider the *d*–*d* interaction between the atoms and the collective decays^2,3,39,40^, and find that the smaller collective decays, the larger QFI can be, when other parameters are the same. What is more, when other parameters are the same, the larger *d*–*d* interaction between the atoms leads to larger QFI at the same moment. These two results can be used in the open system to protect the QFI while there are phase diffusions of PNLs.

Besides, we want to find a general method for calculating the independent phase noise at each qubit of the two-atomic system with d–d interaction. And we have gotten the general numeric solution method of the evolved system density matrix at last.

## Results

### The model

At first we need to introduce QFI. We know that the estimation accuracy of parameter $\phi$ is given by the QFI in the quantum framework, which is^4–7,13,41,42^

1 $$\begin{aligned} \langle {(\Delta \phi )^2}\rangle _\phi \ge \frac{1}{ F_Q(\phi )}. \end{aligned}$$

This is quantum Cramér–Rao theorem, and it is an important theorem of quantum metrology^4–7,13^. It can be seen from this theorem that, under the quantum framework, the accuracy of parameter estimation is determined by the QFI, and the larger the value is, the smaller the variance of the estimator will be Eq. (1) is logically consistent with the Cramér–Rao theorem^42^:

2 $$\begin{aligned} \langle (\Delta \phi )^2\rangle _\phi \ge \frac{1}{F_C(\phi )}. \end{aligned}$$

The difference is in quantum Cramér–Rao theorem (1), we replace the classical Fisher information $F_C (\phi )$ of Cramér–Rao theorem (2) with QFI $F_Q(\phi )$. As $F_Q(\phi )\ge F_C (\phi )$, theorem (1) is the theoretic limit of the accuracy of measuring parameter $\phi$.

By diagonalizing $\rho (\phi )$, the density matrix is

3 $$\begin{aligned} \rho (\phi )=\sum \limits _k\lambda _k|\Psi _k\rangle \langle \Psi _k|, \end{aligned}$$

associated with $\lambda _k\ge 0$ and $\sum \nolimits _k\lambda _k = 1$. The definition of $F_Q(\phi )$ is^4,5,36,42,43^

4 $$\begin{aligned} F_Q(\phi )= & {} Tr[\rho (\phi )L^2_\phi ]=\sum \limits _j\langle \Psi _j|\rho (\phi )L^2_\phi |\Psi _j\rangle =\sum \limits _j\lambda _j \langle \Psi _j|L^2_\phi |\Psi _j\rangle \end{aligned}$$

5 $$\begin{aligned}= & {} \sum \limits _{j,k}\lambda _j\langle \Psi _j|L_\phi | \Psi _k\rangle \langle \Psi _k |L_\phi | \Psi _j\rangle . \end{aligned}$$

Here $L_\phi$ is the ’symmetric logarithmic derivative’ (SLD)^4,36,41,43^ of $\rho (\phi )$, which is a Hermitian operator determined by

6 $$\begin{aligned} \partial _\phi \rho (\phi ) =\frac{\partial {\rho (\phi )}}{\partial \phi } = \frac{1}{2}\{\rho (\phi ), L_\phi \} =\frac{L_\phi \rho (\phi )+\rho (\phi )L_\phi }{2}. \end{aligned}$$

By using Eqs. (6) and (3), $L_\phi$ can be written with^4^

7 $$\begin{aligned} L_\phi =2\sum \limits _{j,k}{\frac{\langle {\Psi _j}|\partial _\phi \rho (\phi )\vert {\Psi _k}\rangle }{\lambda _j +\lambda _k}} \vert {\Psi _j}\rangle \langle {\Psi _k}|. \end{aligned}$$

Substitute this equation into Eq. (5), we can get^4,42^

8 $$\begin{aligned} F_Q(\phi )=\sum \limits _{j,k}{2\frac{\vert \langle {\Psi _j}|\partial _\phi \rho (\phi )\vert {\Psi _k}\rangle \vert ^2}{\lambda _j +\lambda _k}}. \end{aligned}$$

This equation is the general expression of QFI in the diagonalized space of the density matrix $\rho (\phi )$.

Now we begin to discuss the situation where the atoms have the qubit-qubit interactions. For simplicity, we only discuss two qubits (or atoms) interact with two individual PNLs^20,21^ , i.e., each of the two atoms is assumed to be driven by a classical field with a randomly fluctuating phase $\Phi _l(t)$, where $l=1,2$ indicates the two atoms separately. They are the two lasers’ phase noises respectively which act on their respective atoms by the interaction terms. This noise $\Phi _1(t)$ (or $\Phi _2(t)$ ) means laser’s phase will diffuse with time, and the initial phase will no longer be a constant. So the Hamiltonian with the interaction part of the atom and with the phase noise $\Phi _l(t)$ of the PNL is

9 $$\begin{aligned} H=\sum \limits _{l=1}^2 {\lambda [{\hat{\sigma }}_-^l \text{ e}^{i(\Phi _l (t)+\omega t)}+{\hat{\sigma }}_+^l \text{ e}^{-i(\Phi _l (t)+\omega t)}]} +\sum \limits _{l=1}^2 {\frac{1}{2}\omega _{0l} {\hat{\sigma }}_z^l }+g({\hat{\sigma }}_+^1 {\hat{\sigma }}_-^2 +{\hat{\sigma }}_-^1 {\hat{\sigma }}_+^2), \end{aligned}$$

where one of the two atoms with frequency $\omega _{0l}$ is described by the upper state $\vert 0\rangle$ and ground state $\vert 1\rangle$ with $\mathop {{\hat{\sigma }}}\nolimits _+^{1(2)}$ $=\vert 0\rangle _{1(2)} \langle 1\vert$ $$=\left( {\begin{matrix} 0 &{} 1 \\ 0 &{} 0 \\ \end{matrix}}\right) \otimes I_2$$ $(I_2\otimes$ $$\left( {\begin{matrix} 0 &{} 1 \\ 0 &{} 0 \\ \end{matrix}}\right) $$ ), $\mathop {{\hat{\sigma }}}\nolimits _-^{1(2)}=\vert 1\rangle _{1(2)} \langle 0\vert$ $$=\left( {\begin{matrix} 0 &{} 0 \\ 1 &{} 0 \\ \end{matrix}}\right) \otimes I_2$$ $(I_2\otimes$ $$\left( {\begin{matrix} 0 &{} 0 \\ 1 &{} 0 \\ \end{matrix}}\right) $$ ) and $\mathop {{\hat{\sigma }}}\nolimits _z^{1(2)} =\vert 0\rangle _{1(2)} \langle 0\vert -\vert 1\rangle _{1(2)} \langle 1\vert$ $$=\left( {\begin{matrix} 1 &{} 0 \\ 0 &{} -1 \\ \end{matrix}}\right) \otimes I_2$$ $(I_2\otimes$ $$\left( {\begin{matrix} 1 &{} 0 \\ 0 &{} -1 \\ \end{matrix}}\right) $$ )^41^. As we know, if the phase noises are random telegraph noise (RTN), the RTN model means that $\Phi _1(t)$ (or $\Phi _1(t)$) jumps between two values, e.g., *a* and $-a$^1,44,45^, which is a Poisson process. Here we discuss laser phase noise of the standard phase diffusion model, the phase noise $\Phi _l(t)$ is a simple one-dimensional random walk or Wiener process, i.e., $\dot{\Phi }_l(t)$ is white noise: $\langle \langle {{\dot{\Phi }}_l(t)}{{\dot{\Phi }}_l(t+\tau )}\rangle \rangle$ $=2D_l\delta (\tau )$, $l=1,2$. So the atom is effectually being driven by complex colored noise $e^{i\Phi (t)}$ with correlation function

10 $$\begin{aligned} \langle \langle e^{i\Phi _l(t)}e^{-i\Phi _l(t+\tau )}\rangle \rangle =e^{-D_l\tau }, \end{aligned}$$

where subscripts $l=1,2$ indicate the two atoms separately. It is the fact that the driving term is colored noise that ultimately leads to a non-Markovian master equation.

Let us consider the Hamiltonian (9) in the rotating frame. Here we use the unitary transformation with operator $U=\text{ exp }(-\textstyle {i \over 2}\sum \nolimits _{l=1}^2 {\omega _l {\hat{\sigma }}_z^l t})$ to act in the Hamiltonian (9). And under rotating-wave approximation, in the basis of $4\times 4$: $E^{(2)}=$

11 $$\begin{aligned} \begin{aligned} \left( \begin{array}{*{16}c} \vert 00\rangle \langle 00\vert &{} \vert 00\rangle \langle 01\vert &{} \vert 00\rangle \langle 10\vert &{} \vert 00\rangle \langle 11\vert \\ \vert 01\rangle \langle 00\vert &{} \vert 01\rangle \langle 01\vert &{} \vert 01\rangle \langle 10\vert &{} \vert 01\rangle \langle 11\vert \\ \vert 10\rangle \langle 00\vert &{} \vert 10\rangle \langle 01\vert &{} \vert 10\rangle \langle 10\vert &{} \vert 10\rangle \langle 11\vert \\ \vert 11\rangle \langle 00\vert &{} \vert 11\rangle \langle 01\vert &{} \vert 11\rangle \langle 10\vert &{} \vert 11\rangle \langle 11\vert \\ \end{array}\right) , \end{aligned} \end{aligned}$$

two qubits’ Hamiltonian in the rotating frame (i.e., under the unitary transformation of *U*) is

12 $$\begin{aligned} {\mathscr {H}}(\Phi _1,\Phi _2)= & {} \sum \limits _{l=1}^2 {\lambda [{\hat{\sigma }}_-^l \text{ e}^{i\Phi _l (t)}+{\hat{\sigma }}_+^l \text{ e}^{-i\Phi _l (t)}]} +\sum \limits _{l=1}^2 {\frac{1}{2}\Delta _l {\hat{\sigma }}_z^l } +g(\text{ e}^{i(\omega _1 -\omega _2 )t}{\hat{\sigma }}_+^1 {\hat{\sigma }}_-^2 +\text{ e}^{-i(\omega _1 -\omega _2 )t}{\hat{\sigma }}_-^1 {\hat{\sigma }}_+^2) \end{aligned}$$

13 $$\begin{aligned}= & {} \lambda \left( {{\begin{array}{*{20}c} {\frac{\Delta 1+\Delta 2}{2\lambda }} &{} {\text{ e}^{-i\Phi _2 (t)}} &{} {\text{ e}^{-i\Phi _1 (t)}} &{} 0 \\ {\text{ e}^{i\Phi _2 (t)}} &{} {\frac{\Delta 1-\Delta 2}{2\lambda }} &{} {g\,\text{ e}^{i(\omega _1 -\omega _2 )t}/\lambda } &{} {\text{ e}^{-i\Phi _1 (t)}} \\ {\text{ e}^{i\Phi _1 (t)}} &{} {g\,\text{ e}^{-i(\omega _1 -\omega _2 )t}/\lambda } &{} {\frac{-\Delta 1+\Delta 2}{2\lambda }} &{} {\text{ e}^{-i\Phi _2 (t)}} \\ 0 &{} {\text{ e}^{i\Phi _1 (t)}} &{} {\text{ e}^{i\Phi _2 (t)}} &{} {\frac{-\Delta 1-\Delta 2}{2\lambda }} \\ \end{array} }} \right) . \end{aligned}$$

Here we have chosen the same $\lambda$ for the two qubits, and the two qubits are assumed to have the *d*–*d* interaction (or qubit-qubit interaction) with the coefficient *g*. $\Delta _l$ $=\omega _{0l}$ $-\omega _l$ $(l=1,2)$ is the detuning and we assume the two atoms are driven by similar noise lasers (i.e., $\omega _1$ $=\omega _2$ $=\omega$). Below we let $\Delta _1 =\Delta _2 =0$ for simplicity. So we have $\omega _{01}$ $=\omega _{02}$ $=\omega _0$ $=\omega$, which means the transition frequency of the two atoms is resonance with the lasers. For calculating the QFI, our purpose is to obtain the two qubits’ last evolution state $\overline{{\rho }(\phi )}$ which is the stochastic average^39^ of all the possible evolution states. When the atoms are closer, there is collective decay^2,3,40^, and here we use the master equation with collective decay^39^: $\frac{\partial \rho (\Phi _1,\Phi _2,t)}{\partial t}=$ $-i[{\mathscr {H}}(\Phi _1,\Phi _2), \rho (\Phi _1,\Phi _2,t)]$ $-\sum \nolimits _{i,j=1}^2$ ${\gamma _{ij}}$ $(S_+^i S_-^j \rho (\Phi _1,\Phi _2,t) -2S_-^j \rho (\Phi _1,\Phi _2,t) S_+^i$ $+\rho (\Phi _1,\Phi _2,t) S_+^i S_-^j )$. Here $S_\pm ^{1(2)}$ $=$ $\sigma _\pm ^{1(2)}$. Note that the coefficient matrix $(\gamma _{ij})_{ij}$ of the collective decay has not only diagonal but also non-diagonal elements, which are the spontaneous decay rates for the cooperative system^2,40^.

Let us consider how to construct the probability distribution $P(\Phi _1, \Phi _2, t)$. If we assume that in a small time interval $\Delta t$, there is equal probability $R_l\Delta t$ of $\Phi _l$ increasing or decreasing by an amount $\Delta \Phi _l$ then we have

14 $$\begin{aligned} &P(\Phi _1, \Phi _2, t+\Delta t)=(1-(R_1+R_2)\Delta t)P(\Phi _1 ,\Phi _2, t)+\frac{1}{2}R_1\Delta t[P(\Phi _1-\Delta \Phi _1, \Phi _2)\\&\quad +P(\Phi _1+\Delta \Phi _1,\Phi _2, t)]+\frac{1}{2}R_2\Delta t[P(\Phi _1, \Phi _2-\Delta \Phi _2, t)+P(\Phi _1, \Phi _2+\Delta \Phi _2, t)]. \end{aligned} $$

In the limit of $\Delta t\rightarrow 0$, $\Delta \Phi _1 \rightarrow 0$, $\Delta \Phi _2 \rightarrow 0$, $R_1\rightarrow \infty$ and $R_2\rightarrow \infty$ such that

15 $$\begin{aligned} D_1 =\frac{1}{2}R_1(\Delta \Phi _1 )^2 \quad D_2 =\frac{1}{2}R_2(\Delta \Phi _2 )^2 \end{aligned}$$

is the constant diffusion rates of the two PNLs, respectively. And Eq. (14) leads to the well-known probability diffusion equation^31^

16 $$\begin{aligned} \frac{\partial P(\Phi _1, \Phi _2, t)}{\partial t}=D_1 \frac{\partial ^2P(\Phi _1, \Phi _2, t)}{\partial \Phi _1 ^2}+D_2 \frac{\partial ^2P(\Phi _1, \Phi _2, t)}{\partial \Phi _2 ^2}. \end{aligned}$$

Below we will derive the master equation of quantum density operator $\rho (\Phi _1, \Phi _2, t)$ under the evolution of random phase noise $\Phi _l$, $l=1,2$. It will be normalized to unity, i.e., $Tr[\rho (\Phi _1, \Phi _2, t)]=1$. The density operator $\rho (\Phi _1, \Phi _2, t)$ will be the density operator obtained by averaging over all the previous history of evolution that lead to the random variable $\Phi _l$ having the current value $\Phi _l$.For small time interval $\Delta t$ and small phase intervals: $\Delta \Phi _1$ and $\Delta \Phi _2$, the density operator $\rho (\Phi _1, \Phi _2, t+\Delta t)$ will be made up from five contributions depending on whether the $\rho (\Phi _1, \Phi _2, t+\Delta t)$ evolves freely with no change in $\Phi _1$ and $\Phi _2$ with the probability of $(1-(R_1+R_2)\Delta t)$, or else there have occurred four ‘jumps’^31,38^: $\rho (\Phi _1 \pm \Delta \Phi _1, \Phi _2, t)$ $\rightarrow \rho (\Phi _1, \Phi _2, t+\Delta t)$ with the probability of $\frac{1}{2}R_1\Delta t$ for each one, and $\rho (\Phi _1 ,\Phi _2 \pm \Delta \Phi _2, t)$ $\rightarrow \rho (\Phi _1, \Phi _2, t+\Delta t)$ with the probability of $\frac{1}{2}R_2\Delta t$ for each one. The states $\rho (\Phi _1, \Phi _2, t)$, $\rho (\Phi _1 \pm \Delta \Phi _1, \Phi _2, t)$ and $\rho (\Phi _1, \Phi _2 \pm \Delta \Phi _2 ,t)$ have to be separately weighted by the probabilities $P(\Phi _1, \Phi _2 , t)$, $P(\Phi _1 \pm \Delta \Phi _1, \Phi _2, t)$ and $P(\Phi _1, \Phi _2 \pm \Delta \Phi _2, t)$ at time *t*. Putting this all together and normalizing the state yield

17 $$\begin{aligned}  \rho (\Phi _1, \Phi _2, t+\Delta t)=&\{(1+{\mathscr {L}}(\Phi _1, \Phi _2)\Delta t)(1-(R_1+R_2)\Delta t)P(\Phi _1, \Phi _2, t))\rho (\Phi _1,\Phi _2, t) \\&+\frac{1}{2}R_1\Delta tP(\Phi _1-\Delta \Phi _1, \Phi _2, t)\rho (\Phi _1-\Delta \Phi _1,\Phi _2, t) \\&+\frac{1}{2}R_1\Delta t P(\Phi _1+\Delta \Phi _1, \Phi _2, t)\rho (\Phi _1+\Delta \Phi _1, \Phi _2, t)\\&+\frac{1}{2}R_2\Delta t P(\Phi _1, \Phi _2-\Delta \Phi _2, t)\rho (\Phi _1, \Phi _2-\Delta \Phi _2, t)\\&+\frac{1}{2}R_2\Delta t P(\Phi _1, \Phi _2+\Delta \Phi _2, t)\rho (\Phi _1, \Phi _2+\Delta \Phi _1, t)\}\\&\div \{(1-(R_1+R_2)\Delta t)P(\Phi _1, \Phi _2, t)\\&+\frac{1}{2}R_1\Delta t[P(\Phi _1-\Delta \Phi _1 , \Phi _2, t)+P(\Phi _1+\Delta \Phi _1, t, \Phi _2, t)]\\&+\frac{1}{2}R_2\Delta t[P(\Phi _1, \Phi _2-\Delta \Phi _2, t) +P(\Phi _1, \Phi _2+\Delta \Phi _2, t)]\}, \end{aligned} $$

where

18 $$\begin{aligned}  {\mathscr {L}}\text{( }\Phi _1 \text{, }\Phi _2)\rho (\Phi _1,\Phi _2, t)=&-i[{\mathscr {H}}\text{( }\Phi _1 \text{, }\Phi _2),\rho (\Phi _1,\Phi _2, t)] -\gamma (S^+S^-\rho (\Phi _1,\Phi _2, t)\\&-2S^-\rho (\Phi _1,\Phi _2, t) S^+ +\rho (\Phi _1,\Phi _2, t)S^+S^-). \end{aligned} $$

Here we have considered the zero-point fluctuations and 2$\gamma$ is equal to Einstein A coefficient for a single atom and $S^\pm =\sum \nolimits _{l=1}^2 {S^l_\pm }$.

In the limit of $\Delta t\rightarrow 0$, $\Delta \Phi _1 \rightarrow 0$, $\Delta \Phi _2 \rightarrow 0$, $R_1\rightarrow \infty$ and $R_2\rightarrow \infty$, by using Eqs. (14) and (15), Eq. (17) can be transformed into

19 $$\begin{aligned} \frac{\partial [P(\Phi _1, \Phi _2, t)\rho (\Phi _1, \Phi _2 ,t)]}{\partial t}=&{\mathscr {L}}\text{( }\Phi _1 \text{, }\Phi _2)P(\Phi _1, \Phi _2 ,t)\rho \text{( }\Phi _1 \text{, }\Phi _2,t)\\&+D_1 \frac{\partial ^2P(\Phi _1, \Phi _2, t)\rho (\Phi _1, \Phi _2, t)}{\partial \Phi _1 ^2}\\&+D_2 \frac{\partial ^2P(\Phi _1, \Phi _2, t)\rho (\Phi _1, \Phi _2, t)}{\partial \Phi _2^2}. \end{aligned} $$

We assume that the phase $\Phi _1$ and $\Phi _2$ is initially uniformly distributed over [0, $2\pi$), and hence for all time, the probability distribution of the phase $\Phi _1$ and $\Phi _2$ will be its steady state distribution $P\text{( }\Phi _1 \text{, }\Phi _2,t)=\frac{1}{2\pi }$. And we assume the initial condition $\rho \text{( }\Phi _1 \text{, }\Phi _2, 0)=\rho \text{( }0)$, which means the system is brought into interaction with the stochastic influence in a state that is uncorrelated with the stochastic driving term ${\mathscr {H}}(\Phi _1,\Phi _2)$. As the $P\text{( }\Phi _1\text{, }\Phi _2, t)$ is independent of time, Eq. (19) will be simplified to

20 $$\begin{aligned} \frac{\partial \rho (\Phi _1, \Phi _2, t)}{\partial t}={\mathscr {L}}\text{( }\Phi _1 \text{, }\Phi _2)\rho \text{( }\Phi _1 \text{, }\Phi _2, t)+D_1 \frac{\partial ^2\rho (\Phi _1, \Phi _2 ,t)}{\partial \Phi _1 ^2}+D_2 \frac{\partial ^2\rho (\Phi _1, \Phi _2 ,t)}{\partial \Phi _2 ^2}. \end{aligned}$$

If we discretize the $$\rho (\Phi _1,\Phi _2,t)$$ on the stochastic phases $$\Phi _1,\Phi _2$$ and time *t* with discrete values, we can give the relation of subscripts $$i_1$$ and $$i_2$$ with $$\Phi _1$$ and $$\Phi _2$$: $$\Phi _1=(i_1-1 )\Delta \Phi _1$$ and $$\Phi _2=(i_2-1 )\Delta \Phi _2$$. Then the matrices have three kinds of equal expressions by using subscripts $$i_1$$ and $$i_2$$, $$\Phi _1$$ and $$\Phi _2$$ and $$( {i_1-1} )$$ $$\Delta \Phi _1$$ and $$( {i_2-1} )\Delta \Phi _2$$ respectively: $$\rho ^{n}_{i_1,i_2}$$ $$=\rho (\Phi _1,$$ $$\Phi _2,t)$$ $$=\rho ( ( {i_1-1})\Delta \Phi _1,$$ $$( {i_2-1}) \Delta \Phi _2$$, $$n\Delta t)$$, $$\rho ^{n-1}_{i_1,i_2}$$ $$=\rho (\Phi _1,$$ $$\Phi _2, (n-1)\Delta t)$$ $$=\rho ( ( {i_1-1})\Delta \Phi _1,$$ $$( {i_2-1})\Delta \Phi _2$$, $$(n-1)\Delta t)$$, $${\mathscr {L}}_{i_1,i_2}$$ $$={\mathscr {L}}(\Phi _1,$$ $$\Phi _2)$$ $$ =$$ $${\mathscr {L}}( ( {i_1-1} )$$ $$\Delta \Phi _1,$$ $$( {i_2-1} )\Delta \Phi _2)$$ and $${\mathscr {H}}_{i_1,i_2}$$ $$={\mathscr {H}}(\Phi _1,$$ $$\Phi _2)$$ $$ =$$ $${\mathscr {H}}( ( {i_1-1} )$$ $$\Delta \Phi _1,$$ $$( {i_2-1} )\Delta \Phi _2)$$. And these three kind expressions are the same here and after even under later discussions where these matrices are rearranged. So Eq. (20) can be expressed by the finite difference scheme (here we use the implicit scheme of the finite difference scheme):

21 $$\begin{aligned} \frac{\rho ^{n}_{i_1,i_2} -\rho ^{n-1}_{i_1,i_2} }{\Delta t}&={\mathscr {L}}_{i_1,i_2}\rho ^{n}_{i_1,i_2} +D_1 \frac{\rho ^{n}_{i_1+1,i_2} -\rho ^{n}_{i_1,i_2} -(\rho ^{n}_{i_1,i_2} -\rho ^{n}_{i_1-1,i_2} )}{(\Delta \Phi _1) ^2}\\&\quad +D_2 \frac{\rho ^{n}_{i_1,i_2+1} -\rho ^{n}_{i_1,i_2} -(\rho ^{n}_{i_1,i_2} -\rho ^{n}_{i_1,i_2-1} )}{(\Delta \Phi _2) ^2}. \end{aligned} $$

In this equation, *n* is the time discrete index and can be any value of 1, 2, $\ldots$, $n_m$ ($n_m$ is the maximal time index). And for the superscript, $\rho ^{n}$ means the solution of the density matrix at *n* (or the time *t*), and $\rho ^{n_m}$ means the solution of $\rho ^{n}$ at $n=n_m$ (or the final time $t=t_m$). And the density matrix $\rho ^{n-1}$ is the solution of the density matrix at $n-1$ (or the any time $t-\Delta t$), which means $\rho ^{n-1}$ is the one that $\Delta t$ before $\rho ^{n}$. Note that $\rho ^{0}$ means initial time value of the density matrix $\rho ^{n-1}$ at $n=1$ (or the moment $t=0$). And $\rho ^{1}$ means initial time value of the density matrix $\rho ^{n}$ at $n=1$ (which means the moment $t=\Delta t$), and also it can be matrix $\rho ^{n-1}$ at $n=2$ (which also means the moment $t=\Delta t$). This is the base for our iteration calculation later. And $\Delta t=(t_m-0)/n_m$ which is the time step length. Here $\Delta \Phi _1 =({2\pi -0} )/N_1$ and $\Delta \Phi _2 =({2\pi -0} )/N_2$ are the step lengths of diffusion of phase noise, and $N_1$ and $N_2$ are maximal phase index respectively (i.e., $i_1$ and $i_2$ have the maximal values $N_1$ and $N_2$ respectively). In Eq. (21), for the subscripts, we further choose $i_1$ and $i_2$ from 1 to $N_1$ and 1 to $N_2$ as the phase noise indexes, which means the possible values of phase noise $\Phi _1$ and $\Phi _2$, respectively. And then we can get a series of equations, namely, $N_1N_2$ equations. Summarizing these $N_1N_2$ equations, we can get a matrix equation:

22 $$\begin{aligned} &-({\mathscr {L}}_{i_1,i_2}\rho ^{n}_{i_1,i_2})_{1\le i_1\le N_1,1\le i_2\le N_2} \Delta t\\&\qquad \times ((I_{N_1} +2D_1\frac{\Delta t}{\Delta \Phi _1 ^2}I_{N_1}){\otimes I_2^{\otimes 2}}) (\rho ^{n}_{i_1,i_2})_{1\le i_1\le N_1,1\le i_2\le N_2}\\&\qquad -D_1\frac{\Delta t}{\Delta \Phi _1 ^2} \left( \left( \begin{array}{*{16}c} 0 &{} 1 &{} 0 &{} \cdots &{} 0 &{} 1 \\ 1 &{} 0 &{} 1 &{} 0 &{} \cdots &{} 0 \\ 0 &{} 1 &{} 0 &{} 1 &{} \ddots &{} \vdots \\ \vdots &{} 0 &{} 1 &{} 0 &{} \ddots &{} 0 \\ 0 &{} \vdots &{} \ddots &{} \ddots &{} \ddots &{} 1 \\ 1 &{} 0 &{} \cdots &{} 0 &{} 1 &{} 0 \end{array}\right) _{N_1\times N_1} {\otimes I_2^{\otimes 2}}\right) (\rho ^{n}_{i_1,i_2})_{1\le i_1\le N_1,1\le i_2\le N_2} \\&\qquad +2D_2\frac{\Delta t}{\Delta \Phi _2 ^2} (I_{N_1}{\otimes I_2^{\otimes 2}}) (\rho ^{n}_{i_1,i_2})_{1\le i_1\le N_1,1\le i_2\le N_2}\\&\qquad - D_2\frac{\Delta t}{\Delta \Phi _2 ^2}(\rho ^{n}_{i_1,i_2})_{1\le i_1\le N_1,1\le i_2\le N_2} \left( \left( \begin{array}{*{16}c} 0 &{} 1 &{} 0 &{} \cdots &{} 0 &{} 1 \\ 1 &{} 0 &{} 1 &{} 0 &{} \cdots &{} 0 \\ 0 &{} 1 &{} 0 &{} 1 &{} \ddots &{} \vdots \\ \vdots &{} 0 &{} 1 &{} 0 &{} \ddots &{} 0 \\ 0 &{} \vdots &{} \ddots &{} \ddots &{} \ddots &{} 1 \\ 1 &{} 0 &{} \cdots &{} 0 &{} 1 &{} 0 \\ \end{array}\right) _{N_2\times N_2}{\otimes I_2^{\otimes 2}}\right) \\&\quad =(\rho ^{n-1}_{i_1,i_2})_{1\le i_1\le N_1,1\le i_2\le N_2}. \end{aligned} $$

Note that in the summarizing process we have used $\rho ^{n}_{0,i_2}=\rho ^{n}_{N_1,i_2}$ and $\rho ^{n}_{i_1,0}=\rho ^{n}_{i_1,N_2}$, because of $\rho ( -\Delta \Phi _1,$ $( {i_2-1}) \Delta \Phi _2$, $n\Delta t)$ $=\rho ( ( {N_1-1})\Delta \Phi _1,$ $( {i_2-1}) \Delta \Phi _2$, $n\Delta t)$ and $\rho ( ( {i_1-1})\Delta \Phi _1,$ $-\Delta \Phi _2$, $n\Delta t)=\rho ( ( {i_1-1})\Delta \Phi _1,$ $( {N_2-1}) \Delta \Phi _2$, $n\Delta t)$. Thus we can ensure subscripts satisfying $1\le i_1\le N_1$ and $1\le i_2\le N_2$. Here $$I_{N_l}= \left( \begin{array}{*{16}c} 1 &{} 0 &{} \cdots &{} 0 \\ 0 &{} \ddots &{} \ddots &{} \vdots \\ \vdots &{} \ddots &{} \ddots &{} 0 \\ 0 &{} \cdots &{} 0 &{} 1 \\ \end{array}\right) _{N_l\times N_l}$$ with $(l=1,2)$ is an unit matrix with its dimension of $N_l\times N_l$ here and after. $I_2^{\otimes 2}$ is the unit operator of two qubits to each block. This matrix equation can be simply expressed as

23 $$\begin{aligned} -({\mathscr {L}}_{i_1,i_2}\rho ^{n}_{i_1,i_2})_{1\le i_1\le N_1,1\le i_2\le N_2} \Delta t+({\mathbb {A}}\otimes I_2^{\otimes 2}){\mathbb {X}} +{\mathbb {X}}({\mathbb {B}}\otimes I_2^{\otimes 2})={\mathbb {C}}, \end{aligned}$$

where $${\mathbb {A}} =(bI_{N_1})-a_1{\mathscr {D}}_{N_1},$$ $$~{\mathbb {B}} =-a_2{\mathscr {D}}_{N_2}.$$ And here we have given a simplification: $$\left( \begin{array}{*{16}c} 0 &{} 1 &{} 0 &{} \cdots &{} 0 &{} 1 \\ 1 &{} 0 &{} 1 &{} 0 &{} \cdots &{} 0 \\ 0 &{} 1 &{} 0 &{} 1 &{} \ddots &{} \vdots \\ \vdots &{} 0 &{} 1 &{} 0 &{} \ddots &{} 0 \\ 0 &{} \vdots &{} \ddots &{} \ddots &{} \ddots &{} 1 \\ 1 &{} 0 &{} \cdots &{} 0 &{} 1 &{} 0 \\ \end{array}\right) _{N_l\times N_l} ={\mathscr {D}}_{N_l}$$, $$(l = 1,2) $$, in which the two adjacent diagonals of the main diagonal is 1, the matrix element of the top right and bottom left is 1, and other elements are 0. Here $${\mathbb {X}}=(\rho ^{n}_{i_1,i_2} )_{1\le i_1\le N_1,1\le i_2\le N_2}$$ and $${\mathbb {C}}=(\rho ^{n-1}_{i_1,i_2})_{1\le i_1\le N_1,1\le i_2\le N_2}$$ (below we express $$(~~)_{1\le i_1\le N_1,1\le i_2\le N_2}$$ as $$(~~)_{ i_1,i_2}$$ for simplicity). $${\mathbb {X}}$$ and $${\mathbb {C}}$$ are both $$N_1\times N_2$$ block matrices displayed by subscript $$(~~)_{i_1, i_2}$$, and each block is $$4\times 4$$ matrix. Here the first value on moment zero is given by $$\rho (\Phi _1,\Phi _2,0)$$ $$=(\rho ^0_{i_1,i_2})_{1\le i_1\le N_1,1\le i_2\le N_2}$$, which means the first value of *n* is 0. And the last value on moment $$t_m$$ is given by $$\rho (\Phi _1,\Phi _2, t_m)=(\rho ^{n_m}_{i_1,i_2} )_{1\le i_1\le N_1,1\le i_2\le N_2}$$, which means $$(n_m+1)$$th value of *n* is $$n_m$$. And $$b=$$ $$(1+2a_1+2a_2)$$, $$a_1=D_1\frac{\Delta t}{\Delta \Phi _1 ^2}$$ and $$a_2 =D_2\frac{\Delta t}{\Delta \Phi _2 ^2}$$. Here $${\mathbb {X}}$$ and $${\mathbb {C}}$$ are the matrixes to be calculated and $${\mathbb {A}}$$ and $${\mathbb {B}}$$ are constant matrixes.

If we do not consider the item ${\mathscr {L}}_{i_1,i_2}\rho ^{n}_{i_1,i_2}$ in Eq. (21) which contains Hamiltonian, *d*–*d* interaction and the cooperative dissipation, this equation is simple two-dimension phase diffusion matrix equation. But now Eq. (21) contains this item, and it is not a simple phase diffusion matrix equation anymore. That is to say, if we do not consider the first item of Eq. (23), Eq. (23) will actually be Sylvester equation^46^.

### The first rearrangement

Now let us solve Eq. (23). Here we use the way of matrix rearranging in solving the Sylvester equation^46^. At first we need to rearrange the two $N_1 \times N_2$ block matrices: $(\rho ^{n}_{i_1,i_2})_{i_1,i_2}$ and $(\rho ^{n-1}_{i_1,i_2})_{i_1,i_2}$, column block by column block, into two $N_1N_2\times 1$ column block matrices $({\rho }^{n}_{i_2;i_1,1})_{1\le i_2\le N_1;1\le i_1\le N_2,1}$ and $({\rho }^{n-1}_{i_2;i_1,1})_{1\le i_2\le N_1;1\le i_1\le N_2,1}$, and here we use $(~~)_{1\le i_2\le N_1;1\le i_1\le N_2,1}$ to indicate the rearranged block element with column style (below we will express it as $(~~)_{i_2;i_1,1}$ for simplicity). Each block of ${\rho }^{n}_{i_2;i_1,1}$ and ${\rho }^{n-1}_{i_2;i_1,1}$ is still $4\times 4$ matrix. According to this definition of the rearranging, $N_1\times N_2$ block matrix: $({\mathscr {L}}_{i_1,i_2}\rho ^{n}_{i_1,i_2})_{i_1,i_2}$ is rearranged into $N_1N_2\times 1$ column block matrix $({{\mathscr {L}}}_{i_2;i_1,1}\rho ^{n}_{i_2;i_1,1})_{i_2;i_1,1}$ (the blocks of the latter are just the rearrangement of the blocks of the former, column block by column block). And indexed by $i_1$ and $i_2$, each block of ${{\mathscr {L}}}_{i_2;i_1,1}\rho ^{n}_{i_2;i_1,1}$, ${\rho }^{n}_{i_2;i_1,1}$ and ${\rho }^{n-1}_{i_2;i_1,1}$ is the simply expression of ${{\mathscr {L}}}_{(i_2-1) N_1+(i_1-1),1}{\rho }^{n}_{(i_2-1) N_1+(i_1-1),1}$, ${\rho }^{n}_{(i_2-1) N_1+(i_1-1),1}$ and ${\rho }^{n-1}_{(i_2-1) N_1+(i_1-1),1}$, which is actually the block elements of the former matrix to be rearranged: ${\mathscr {L}}_{i_1,i_2}\rho ^{n}_{i_1,i_2}$, $\rho ^{n}_{i_1,i_2}$ and $\rho ^{n-1}_{i_1,i_2}$, respectively. Here in $(~~)_{i_2;i_1,1}$ we change the order of $i_1$ and $i_2$ because the rearrangement is column block by column block and even index $i_2$ gives more priority order than odd index $i_1$. This can be seen from the rearranging sequence value $(i_2-1) N_1+(i_1-1)$. And the block subscripts of $(~~)_{i_2;i_1,1}$ mean the block rearranging sequence by which we can rearrange the blocks inside $(~~)_{i_1,i_2}$ under the sequence value $(i_2-1) N_1+(i_1-1)$ from 0 to $N_1N_2-1$.

Under this first rearrangement, the second item and the third item of Eq. (23) can be rearranged into $(I_{N_2}$ $\otimes {{\mathbb {A}}}\otimes I_2^{\otimes 2})~{\mathbb {X}}$ and $({{\mathbb {B}}}^T$ $\otimes I_{N_1}\otimes I_2^{\otimes 2})$ ${\mathbb {X}}$, respectively. Then Eq. (23) can be changed into

24 $$\begin{aligned}&-({{\mathscr {L}}}_{i_2;i_1,1}{\rho }^{n}_{i_2;i_1,1})_{i_2;i_1,1} \Delta t+(I_{N_2} \otimes {\mathbb {A}}\otimes I_2^{\otimes 2})(\rho ^n_{i_2;i_1,1})_{i_2;i_1,1} \nonumber \\&\quad +({\mathbb {B}}^T\otimes I_{N_1}\otimes I_2^{\otimes 2})(\rho ^n_{i_2;i_1,1})_{i_2;i_1,1}=(\rho ^{n-1}_{i_2;i_1,1})_{i_2;i_1,1}, \end{aligned}$$

Because each block is not changed before and after rearranging, i.e. ${\mathscr {L}}_{i_2;i_1,1}\rho ^{n}_{i_2;i_1,1}$ $=$ ${\mathscr {L}}_{i_1,i_2}\rho ^{n}_{i_1,i_2}$; ${\mathscr {H}}_{i_2;i_1,1}$ $=$ ${\mathscr {H}}_{i_1,i_2}$; $\rho ^{n}_{i_2;i_1,1}$ $=$ $\rho ^{n}_{i_1,i_2}$; $\rho ^{n-1}_{i_2;i_1,1}$ $=$ $\rho ^{n-1}_{i_1,i_2}$, after rearranging we have

25 $$\begin{aligned} {\mathscr {L}}_{i_2;i_1,1}\rho ^{n}_{i_2;i_1,1}=-i[{\mathscr {H}}_{i_2;i_1,1},\rho ^n_{i_2;i_1,1}]-\gamma (S^+S^-\rho ^{n}_{i_2;i_1,1} -2S^-\rho ^{n}_{i_2;i_1,1}S^++\rho ^{n}_{i_2;i_1,1}S^+S^-), \end{aligned}$$

which is still Eq. (18), only expressed under rearranging subscripts of the block matrixes. From Eq. (25), we know that each block ${{\mathscr {L}}}_{i_2;i_1,1}{\rho }^{n}_{i_2;i_1,1}$ of $({{\mathscr {L}}}_{i_2;i_1,1}{\rho }^{n}_{i_2;i_1,1})_{i_2;i_1,1}$ is a whole block matrix and can not be separated into two independent matrices (i.e. $4\times 4$ matrix ${{\mathscr {L}}}_{i_2;i_1,1}$ and $4\times 4$ matrix ${\rho }^{n}_{i_2;i_1,1}$) multiplying directly. Thus the entirety matrix $({{\mathscr {L}}}_{i_2;i_1,1}{\rho }^{n}_{i_2;i_1,1})_{i_2;i_1,1}$ also can not be separated into two block matrixes multiplexing, so to separate the entirety matrix $({{\mathscr {L}}}_{i_2;i_1,1}{\rho }^{n}_{i_2;i_1,1})_{i_2;i_1,1}$, below we will proceed a further rearrangement of Eq. (24).

### The second rearrangement

If we rearrange the basis of the original $$4\times 4$$ basis (i.e., $${E}^{(2)})$$ of $${\mathscr {L}}_{i_2;i_1,1}\rho ^{n}_{i_2;i_1,1}$$ in Eq. (24) row element by row element into $${\tilde{E}}^{(2)}=$$ $$(\vert 00\rangle \langle 00\vert $$, $$\vert 00\rangle \langle 01\vert $$, $$\vert 00\rangle \langle 10\vert $$, $$\vert 00\rangle \langle 11\vert $$, $$\vert 01\rangle \langle 00\vert $$, $$\vert 01\rangle \langle 01\vert $$, $$\vert 01\rangle \langle 10\vert $$, $$\vert 01\rangle \langle 11\vert $$, $$\vert 10\rangle \langle 00\vert $$, $$\vert 10\rangle \langle 01\vert $$, $$\vert 10\rangle \langle 10\vert $$, $$\vert 10\rangle \langle 11\vert $$, $$\vert 11\rangle \langle 00\vert $$, $$\vert 11\rangle \langle 01\vert $$, $$\vert 11\rangle \langle 10\vert $$, $$\vert 11\rangle \langle 11\vert )^T$$ $$=(e^1_{00}e^2_{00}$$, $$e^1_{00}e^2_{01}$$, $$e^1_{00}e^2_{10}$$, $$e^1_{00}e^2_{11}$$, $$e^1_{00}e^2_{01}$$, $$e^1_{00}e^2_{01}$$, $$e^1_{01}e^2_{10}$$, $$e^1_{01}e^2_{11}$$, $$e^1_{10}e^2_{00}$$, $$e^1_{10}e^2_{01}$$, $$e^1_{10}e^2_{10}$$, $$e^1_{10}e^2_{11}$$, $$e^1_{11}e^2_{00}$$, $$e^1_{11}e^2_{01}$$, $$e^1_{11}e^2_{10}$$, $$e^1_{11}e^2_{11} )^T$$ which is a $$16\times 1$$ basis that comes from rearranging , then we will get the separated $$\tilde{{\mathscr {L}}}_{i_2;i_1,i_2;i_1}$$ and the separated $${\tilde{\rho }}^{n}_{i_2;i_1,1}$$ multiplying: $$\tilde{{\mathscr {L}}}_{i_2;i_1,i_2;i_1}{\tilde{\rho }}^{n}_{i_2;i_1,1}$$ (column element by column element is also permissible, but the value of the separated $$\tilde{{\mathscr {L}}}_{i_2;i_1,i_2;i_1}$$ and the separated $${\tilde{\rho }} ^{n}_{i_2;i_1,1}$$ will be different from here). Here $$\tilde{{\mathscr {L}}}_{i_2;i_1,i_2;i_1}$$ is a $$16\times 16$$ matrix when $$\rho ^n_{i_2;i_1,1}$$ is rearranged as $${\tilde{\rho }}^{n}_{i_2;i_1,1}$$. Each block, i.e. $${\tilde{\rho }}^{n}_{i_2;i_1,1}$$ or $${\tilde{\rho }}^{n-1}_{i_2;i_1,1}$$, is then $$16\times 1$$ matrix, and the block matrices $$({\tilde{\rho }}^{n}_{i_2;i_1,1})_{i_2;i_1,1}$$ and $$({\tilde{\rho }}^{n-1}_{i_2;i_1,1})_{i_2;i_1,1}$$ are called $$vec({\mathbb {X}})$$ and $$vec({\mathbb {C}})$$ respectively. Throughout our paper, superscript *T* means transposition. Under this matrix rearranging, like the way in solving the Sylvester equation, Eq. (25) can be changed into

26 $$\begin{aligned} \tilde{{\mathscr {L}}}_{i_2;i_1,i_2;i_1}{\tilde{\rho }}^{n}_{i_2;i_1,1}=&-i({\mathscr {H}}_{i_2;i_1,1}\otimes I_2^{\otimes 2} -I_2^{\otimes 2}\otimes {\mathscr {H}}_{i_2;i_1,1}^T){\tilde{\rho }}^{n}_{i_2;i_1,1}\\&-\gamma ((S^+S^-)\otimes I_2^{\otimes 2}-2(S^-\otimes I_2^{\otimes 2})(I_2^{\otimes 2}\otimes (S^+)^T) +I_2^{\otimes 2}\otimes (S^+S^-)^T){\tilde{\rho }}^{n}_{i_2;i_1,1}. \end{aligned}$$

Setting the value $A_{i_l} =\text{ e}^{-i\Phi _l }=e^{-i({i_l-1})*\Delta \Phi _l}$, $A_{i_l}^*=\text{ e}^{i\Phi _l}=e^{i({i_l-1})*\Delta \Phi _l}$, $(l = 1,2)$, $\Gamma =\gamma /\lambda$, $B=-i\Gamma -g/\lambda$, $C=-i\Gamma +g/\lambda$, by some calculations on Eq. (26), we can get $\tilde{{\mathscr {L}}}_{i_2;i_1,i_2;i_1}$ $=-i\lambda \times$

27 $$\begin{aligned} \left( \begin{array}{@{}*{20}{c}@{}} {-4i\Gamma } &{} {-A_{i_2}^*} &{} {-A_{i_1}^*} &{} 0 &{} {A_{i_2} } &{} 0 &{} 0 &{} 0 &{} {A_{i_1} } &{} 0 &{} 0 &{} 0 &{} 0 &{} 0 &{} 0 &{} 0 \\ {-A_{i_2} } &{} {-3i\Gamma } &{} {B } &{} {-A_{i_1}^*} &{} 0 &{} {A_{i_2} } &{} 0 &{} 0 &{} 0 &{} {A_{i_1} } &{} 0 &{} 0 &{} 0 &{} 0 &{} 0 &{} 0 \\ {-A_{i_1} } &{} {B } &{} {-3i\Gamma } &{} {-A_{i_2}^*} &{} 0 &{} 0 &{} 
{A_{i_2} } &{} 0 &{} 0 &{} 0 &{} {A_{i_1} } &{} 0 &{} 0 &{} 0 &{} 0 &{} 0 \\ 0 &{} {-A_{i_1} } &{} {-A_{i_2} } &{} {-2i\Gamma } &{} 0 &{} 0 &{} 0 &{} {A_{i_2} } &{} 0 &{} 0 &{} 0 &{} {A_{i_1} } &{} 0 &{} 0 &{} 0 &{} 0 \\ {A_{i_2}^*} &{} 0 &{} 0 &{} 0 &{} {-3i\Gamma } &{} {-A_{i_2}^*} &{} {-A_{i_1}^*} &{} 0 &{} {C } &{} 0 &{} 0 &{} 0 &{} {A_{i_1} } &{} 0 &{} 0 &{} 0 \\ {2i\Gamma } &{} {A_{i_2}^*} &{} 0 &{} 0 &{} {-A_{i_2} } &{} {-2i\Gamma } &{} {B } &{} {-A_{i_1}^*} &{} 0 &{} {C } &{} 0 &{} 0 &{} 0 &{} {A_{i_1} } &{} 0 &{} 0 \\ {2i\Gamma } &{} 0 &{} {A_{i_2}^*} &{} 0 &{} {-A_{i_1} } &{} {B } &{} {-2i\Gamma } &{} {-A_{i_2}^*} &{} 0 &{} 0 &{} {C } &{} 0 &{} 0 &{} 0 &{} {A_{i_1} } &{} 0 \\ 0 &{} {2i\Gamma } &{} {2i\Gamma } &{} {A_{i_2}^*} &{} 0 &{} {-A_{i_1} } &{} {-A_{i_2} } &{} {-i\Gamma } &{} 0 &{} 0 &{} 0 &{} {C } &{} 0 &{} 0 &{} 0 &{} {A_{i_1} } \\ {A_{i_1}^*} &{} 0 &{} 0 &{} 0 &{} {C } &{} 0 &{} 0 &{} 0 &{} {-3i\Gamma } &{} {-A_{i_2}^*} &{} {-A_{i_1}^*} &{} 0 &{} {A_{i_2} } &{} 0 &{} 0 &{} 0 \\ {2i\Gamma } &{} {A_{i_1}^*} &{} 0 &{} 0 &{} 0 &{} {C } &{} 0 &{} 0 &{} {-A_{i_2} } &{} {-2i\Gamma } &{} {B } &{} {-A_{i_1}^*} &{} 0 &{} {A_{i_2} } &{} 0 &{} 0 \\ {2i\Gamma } &{} 0 &{} {A_{i_1}^*} &{} 0 &{} 0 &{} 0 &{} {C } &{} 0 &{} {-A_{i_1} } &{} {B } &{} {-2i\Gamma } &{} {-A_{i_2}^*} &{} 0 &{} 0 &{} {A_{i_2} } &{} 0 \\ 0 &{} {2i\Gamma } &{} {2i\Gamma } &{} {A_{i_1}^*} &{} 0 &{} 0 &{} 0 &{} {C } &{} 0 &{} {-A_{i_1} } &{} {-A_{i_2} } &{} {-i\Gamma } &{} 0 &{} 0 &{} 0 &{} {A_{i_2} } \\ 0 &{} 0 &{} 0 &{} 0 &{} {A_{i_1}^*} &{} 0 &{} 0 &{} 0 &{} {A_{i_2}^*} &{} 0 &{} 0 &{} 0 &{} {-2i\Gamma } &{} {-A_{i_2}^*} &{} {-A_{i_1}^*} &{} 0 \\ 0 &{} 0 &{} 0 &{} 0 &{} {2i\Gamma } &{} {A_{i_1}^*} &{} 0 &{} 0 &{} {2i\Gamma } &{} {A_{i_2}^*} &{} 0 &{} 0 &{} {-A_{i_2} } &{} {-i\Gamma } &{} {B } &{} {-A_{i_1}^*} \\ 0 &{} 0 &{} 0 &{} 0 &{} {2i\Gamma } &{} 0 &{} {A_{i_1}^*} &{} 0 &{} {2i\Gamma } &{} 0 &{} {A_{i_2}^*} &{} 0 &{} {-A_{i_1} } &{} {B } &{} {-i\Gamma } &{} {-A_{i_2}^*} \\ 0 &{} 0 &{} 0 &{} 0 &{} 0 &{} {2i\Gamma } &{} {2i\Gamma } &{} {A_{i_1}^*} &{} 0 &{} {2i\Gamma } &{} {2i\Gamma } &{} {A_{i_2}^*} &{} 0 &{} {-A_{i_1} } &{} {-A_{i_2} } &{} 0 \\ \end{array} \right) . \end{aligned}$$

Furthermore, following the sequence $(~~)_{i_2;i_1,1}$ of the first block rearrangement, the second rearranged block matrix: $(\tilde{{\mathscr {L}}}_{i_2;i_1,i_2;i_1})_{i_2;i_1,i_2;i_1}$, is a $N_1N_2\times N_1N_2$ block diagonal matrix, and the diagonal block matrices are $\tilde{{\mathscr {L}}}_{i_2;i_1,i_2;i_1}$. So there are $N_1N_2$ diagonal blocks in the second rearranged matrix $(\tilde{{\mathscr {L}}}_{i_2;i_1,i_2;i_1})_{i_2;i_1,i_2;i_1}$. Thus the first item of Eq. (23), i.e. $({\mathscr {L}}_{i_2;i_1,i_2;i_1}\rho ^{n}_{i_2;i_1,i_2;i_1})_{i_2;i_1,i_2;i_1}$, has been separated into two rearranged matrices $(\tilde{{\mathscr {L}}}_{i_2;i_1,i_2;i_1})_{i_2;i_1, i_2;i_1}$ and $vec({\mathbb {X}})$ multiplexing, and the second item and the last item of Eq. (23) can be rearranged into $(I_{N_2}$ $\otimes {{\mathbb {A}}}\otimes I_2^{\otimes 4})~vec({\mathbb {X}})$ and $({{\mathbb {B}}}^T$ $\otimes I_{N_1}\otimes I_2^{\otimes 4})$ $vec({\mathbb {X}})$, respectively.

So after this second rearrangement, Eq. (24) can be transformed into

28 $$\begin{aligned} (-(\tilde{{\mathscr {L}}}_{i_2;i_1,i_2;i_1})_{i_2;i_1,i_2;i_1}+I_{N_2} \otimes {\mathbb {A}}\otimes I_2^{\otimes 4} +{\mathbb {B}}^T\otimes I_{N_1}\otimes I_2^{\otimes 4})vec({\mathbb {X}})=vec({\mathbb {C}}), \end{aligned}$$

where $vec({\mathbb {X}})$ or $vec({\mathbb {C}})$ comes from rearranging its own $N_1\times N_2$ square block matrix (i.e., ${\mathbb {X}}$ or ${\mathbb {C}}$) column block by column block into $N_1N_2$ $\times$ 1 column block matrix indexed with subscript ${i_2;i_1,1}$ (i.e., $(~~)_{i_2;i_1,1}$, which is arranged by the sequence value of $(i_2-1) N_1+(i_1-1)$ from 0 to $N_1N_2-1$ just as we have defined), and each block is still $16\times 1$. The detail definitions about the basis of matrices $vec({\mathbb {X}})$, $vec({\mathbb {C}})$ and $(\tilde{{\mathscr {L}}}_{i_2;i_1,i_2;i_1})_{i_2;i_1,i_2;i_1}$ are in the Supplementary Information 1. Equation (28) can be calculated by the iterations with

29 $$\begin{aligned} vec({\mathbb {X}})=(-(\tilde{{\mathscr {L}}}_{i_2;i_1,i_2;i_1})_{i_2;i_1,i_2;i_1}+I_{N_2}\otimes {\mathbb {A}}\otimes I_2^{\otimes 4}+{\mathbb {B}}^T\otimes I_{N_1}\otimes I_2^{\otimes 4})^{-1}vec({\mathbb {C}}). \end{aligned}$$

This equation can give us the convenience of the iteration calculations. Here the initial value $$vec({\mathbb {C}})$$ $$=$$ $$({\tilde{\rho }}^0_{i_2;i_1,1})_{i_2;i_1,1} $$ is $$N_1N_2$$ blocks, and each block is the same. Below we provide each block is $${\tilde{\rho }}^0_{i_2;i_1,1} =(\cos ^2\theta $$,0,0, $$\cos \theta \sin \theta e^{-2i\phi }$$, 0,0,0,0,0,0,0,0, $$\cos \theta \sin \theta e^{2i\phi }$$, 0,0, $$\sin ^2\theta )^T$$ for any $$1\le i_2\le N_2; 1\le i_1\le N_1$$, which is the matrix of the two qubit maximal entangled state $$\vert \Psi ^0\rangle =\cos \theta \vert 00\rangle $$ $$+\sin \theta \exp (i2\phi )\vert 11\rangle $$. And we provide $$\theta =\pi /4$$ and $$\phi =\pi /5$$ throughout this paper. Note that this $$\phi $$ is a fixed phase value at the initial time, and it is to be estimated which has no relation with the former phase noise, i.e. $$\Phi _1$$ and $$\Phi _2$$. We can use the program to iterate $$n_m$$ times between $$vec({\mathbb {C}})$$ and $$vec({\mathbb {X}})$$, i.e., one iteration result $$vec({\mathbb {X}})$$ is used as the initial value $$vec({\mathbb {C}})$$ of the next iteration. In the end we can get the result, a set constituted from a series of $$vec({\mathbb {X}})$$, namely $$\{({\tilde{\rho }}^n_{i_2;i_1,1})_{i_2;i_1,1}\}$$ $$(1\leqslant n\leqslant n_m)$$. Adding the initial state $$({\tilde{\rho }}^0_{i_2;i_1,1})_{i_2;i_1,1}$$ into this set, we get a new set $$\{({\tilde{\rho }}^n_{i_2;i_1,1})_{i_2;i_1,1}\}$$ for every moment, where *n* is expand to $$\{0\leqslant n\leqslant n_m\}$$. For each *n*, each $${\tilde{\rho }}^n_{i_2;i_1,1}$$, which means with fixed $$i_2$$ and $$i_1$$, indicates each evolving result according to its possible initial phase noises $$\Phi _2$$ and $$\Phi _1$$ in Hamiltonian of Eq. (9), respectively. Thus for each *n*, $$({\tilde{\rho }}^n_{i_2;i_1,1})_{i_2;i_1,1}$$ has $$N_1N_2$$ independent solutions, or $$N_1N_2$$ block matrices, and each matrix is $$16\times 1$$. We retransform each of these $$N_1N_2$$ block matrices from $$16\times 1$$ matrix back into $$4\times 4$$ matrix $$\{({\rho }^n_{i_2;i_1,1})_{i_2;i_1,1}\}$$ and take the stochastic average^28,44,45,47^ by summing them up and then dividing the summation with $$N_1N_2$$ (these stochastic average is based on that every initial noise possessing any stochastic value in $$[0, 2\pi 
)$$ has equal probability for each qubit). Then we get a series of stochastic averaged density matrixes with discrete form: $$\{{\overline{\rho }}^n\}$$, $$(0\leqslant n\leqslant n_m)$$. $${\overline{\rho }}^n$$ is a $$4\times 4$$ matrix which is the evolved matrix at the moment *t*. Ultimately, we get the evolved matrix: $$\{{\overline{\rho }}^n\}$$, $$(0\leqslant n\leqslant n_m)$$ by our general numeric solution method.

Hence, by using $\{{\overline{\rho }}^n\}$, we can calculate the QFI of two interacting atoms driven by PNLs. By using Eq. (8)^4,42^,

30a $$\begin{aligned} F^n_Q(\phi )&=Tr[\overline{{\rho }(\phi )}^n L^2_\phi ]=\sum \limits _{j,k}{\frac{2|{\langle {\Psi ^n_j} |\frac{\partial {\overline{\rho (\phi )}}^n}{\partial \phi }|\Psi ^n_k\rangle }|^2}{\lambda ^n_j +\lambda ^n_k}}.\\ \end{aligned} $$

It is to calculate the time discrete form of QFI where the time superscript *n* obeys $(0\leqslant n\leqslant n_m)$. Here $\lambda ^n_{j(k)}$ and $|\Psi ^n_{j(k)}\rangle$ are eigenvalue and eigenvector of ${\overline{\rho (\phi )}}^n$ respectively. Note that in Eq. (30a) we need to numerically calculate the derivative of the evolved matrix ${\overline{\rho (\phi )}}^n$ to the estimated phase $\phi$: $\frac{\partial {\overline{\rho (\phi )}}^n}{\partial \phi }$. If we change $\phi$ of $\rho ^0_{i_2;i_1,1}$ into $\phi$ $+$ $\delta \phi$, then using the same way of calculating ${\overline{\rho (\phi )}}^n$ in this paper, we get a evolved matrix ${\overline{\rho (\phi + \delta \phi )}}^n$, and the numeric derivative $\frac{\partial {{\overline{\rho (\phi )}}^n}}{\partial \phi }$ $=$ $\frac{{\overline{\rho (\phi + \delta \phi )}}^n -{\overline{\rho (\phi )}}^n}{\delta \phi }$. In the calculation of this paper, we as well choose $\delta \phi =0.0001$ (it can be tested mathematically, usually $\delta \phi$ $\sim$ $0.0001\phi$ , which gives us a better numerical derivative), then we can get the derivative of ${\overline{\rho (\phi )}}^n$ to the estimated phase $\phi$. And subscribe $n_m+1$ derivatives into Eq. (30a), we can get the QFI of the time discrete form of two atoms driven by the PNLs: $\{F^n_Q (\phi )\}$, $(0\leqslant n\leqslant n_m)$.

### Discussion of the QFI

Below we use $\lambda$ to scale time, i.e., use $\lambda t$ to represent *t*, i.e., we adopt this style in scaling time, which leads to the replacements in above formulas: $\omega$ $\rightarrow$ $\omega /\lambda$, $\gamma$ $\rightarrow$ $\gamma /\lambda$, *D* $\rightarrow$ $D/\lambda$. And for simplicity, we provide the two diffusion coefficients are equal, i.e. $D_1=D_2=D$, and provide $N_1=N_2=N$. And thus we have $a_1 =a_2$.

**Figure 1:**
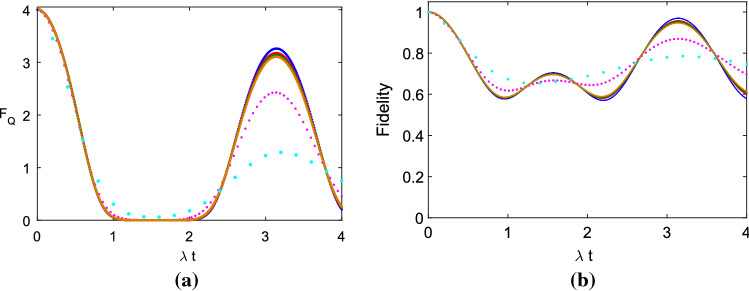
(**a**) QFI vs. $\lambda t$, (**b**) Fidelity vs. $\lambda t$, for $N=34$ and $n_m=903$ (red), $N=28$ and $n_m=613$ (green), $N=25$ and $n_m=488$ (brown), $N=10$ and $n_m=78$ (pink), $N=5$ and $n_m=20$ (sky blue). Here $D/\lambda$ $=0.01$ and the maximal time is $\lambda t_m$ $=4$. Here we do not consider the *d*–*d* interaction and the collective decay of the atoms, i.e., $g=$ $\gamma =0$, and the difference interval of the estimated phase is $\delta \phi$ $=0.0001$. In (**a**,**b**), blue lines indicate the curve got from the analytical expression, and the red, green, brown, pink, sky blue lines indicate the numerical simulation result got from this paper under the same parameters, respectively.

Figure 1 indicates QFI and fidelity of two atoms driven by PNLs under the rotating frame, and here we provide $D/\lambda$ $=0.01$, the maximal time $\lambda t_m=4$ when we use the computers to do calculations. And here we do not consider the *d*–*d* interaction and the collective decay (i.e., $g=$ $\gamma =0$) of the two atoms. And $\phi$ $=\pi /5$ is the estimated phase (actually QFI has not relation with this value), and the difference interval of the estimated phase is $\delta \phi$ $=0.0001$. We divide [0, $2\pi )$ into *N* uniformly spaced values, i.e., *N* is the division number of equally dividing $[0,2\pi )$, or the maximal phase noise index in the former derivation of this paper. From the stochastic viewpoint, all values among $[0,2\pi )$ have the equal chances to be the initial phase noises, and what is more these phase noises will diffuse with time between these *N* values $\{({i-1})$ $\Delta \Phi \}$, $i=1, 2, ..., N$. Actually, strictly speaking, $2\pi$ needs to be divided into infinite values, which is strict correct, but Limited by the calculation abilities of computers, *N* can not be too large.

In (a) and (b) of Fig. 1, blue lines are based on the analytical expression of our former work^1^, i.e. the analytical result, and red, green, brown, pink, sky blue lines are the numeric simulation results based on the method of this paper under the same parameters. For the numeric simulation, from Eqs. (15) and (16), we know that when $D/\lambda$ $=0.01$ is a provided value, $\frac{(\Delta \Phi ) ^2}{\Delta t}$ needs to be concrete value, which means $N^2/n_m$ will be equal in different simulations. So we can provide $N=34$ and $n_m=903$ (red), $N=28$ and $n_m=613$ (green), $N=25$ and $n_m=488$ (brown), $N=10$ and $n_m=78$ (pink), $N=5$ and $n_m=20$ (sky blue) in Fig. 1a,b. From Fig. 1a, the numerical simulation (red, green, brown, pink or sky blue line) of this paper is close to the analytical result (blue line) of our former work^1^. However, different divided values *N* reflect the different numeric simulation accuracies on the calculations of the diffusions of two atoms’ phase noises. Comparing the red, green, brown, pink, sky blue lines with blue lines in (a) and (b), we find larger *N* leads to closer results between the numeric simulation result and the analytical result. This is reasonable for us to understand. And this is consistent with the general law of finite element method for numerical solution of physical model, that is, the smaller the spatial step and the time step, the more accurate the simulation results. Because of the phase diffusion rate $D/\lambda \ne 0$, the QFI and the quantum fidelity will be actually a damping oscillation if we consider a long time evolution.

Below we discuss the situation where *d*–*d* interaction and collective decay exist^2,39,40,47,48^, i.e., *g* and $\gamma$ are not zero. According to the definition of *d*–*d* interaction^2,40,49^, $g_{jk}$ $=$ $\frac{3}{2}\gamma \{(1-3\cos ^2\vartheta )[\sin (k_0r_{jk})/(k_0r_{jk})^2$ $+$ $\cos (k_0r_{jk})/{(k_0r_{jk})}^{3}]$ − $(1-\cos ^2\vartheta )[\cos ({k}_{0}{r}_{jk})/({k}_{0}{r}_{jk})]\}$ and $\gamma _{jk}$ $=$ $\gamma \{\sin (k_0r_{jk})/(k_0r_{jk})$ $+$ $\frac{1}{2}(3\cos ^2\vartheta -1)$ $[(3/(k_0r_{jk})^2$ $-1)\sin (k_0r_{jk})/(k_0r_{jk})$ − $3\cos (k_0r_{jk})/{(k_0r_{jk})}^{2}]\}$ for $j,k=1,2$ and $j\ne k$. Here $k_0$ $=$ $\omega /c$ $=$ $2\pi /s_0$, $s_0$ is the resonant wavelength, the diagonal elements $2\gamma _{11}=2\gamma _{22}=2\gamma =4|{\mathbf {d}_{eg}}|^2\omega _0^3/(3\hbar c^3)$ is just the spontaneous emission coefficient of a single atom, and $\mathbf {d}_{eg}$ is the dipole moment^40^. $\vartheta$ is the angle between the direction of the dipole moment and the line joining the *j*th and the *k*th atom, whose distance is denoted by $r_{jk}$ $=|\overrightarrow{r}_j-\overrightarrow{r}_k|$. From this definition of $g_{jk}$ and $\gamma _{jk}$, it is easy to find $g_{12}=g_{21}=g$ and and $\gamma _{12}=\gamma _{21}$.

For large interatomic separations $k_0r_{jk}$ goes to infinity, then $g_{jk}=\gamma _{jk}=0$ for $j\ne k$, i.e., there is no coupling between the atoms. And their decay will be independent decay. This case can be solved analytically, as we and other authors have done before^1,32–34^.

**Figure 2:**
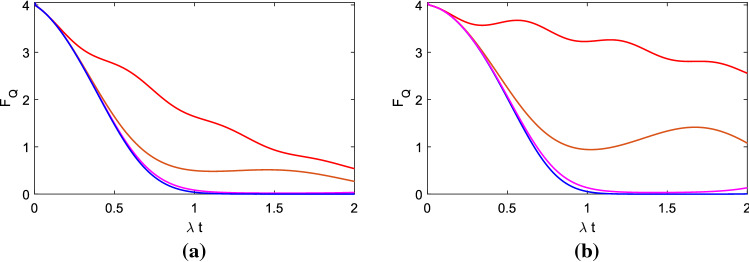
QFI vs. $\lambda t$. Here we provide $D/\lambda$ $=0.01$, $g/\lambda =0$ (blue), $g/\lambda$ $=1$ (pink), $g/\lambda$ $=2.5$ (brown) and $g/\lambda$ $=10$ (red). (**a**) $\gamma /\lambda$ $=\frac{1}{4}$; (**b**) $\gamma /\lambda$ $=\frac{1}{20}$.

When the two atoms are closer, i.e., $k_0r_{jk}$ is near zero, from the above definitions of $g_{jk}$ and $\gamma _{jk}$, we obtain (provide two atomic dipole moments are parallel to each other)^2,49^

31 $$\begin{aligned} g_{jk}=g\approx \frac{3\gamma }{2(k_0r_{jk})^3}(1-3\cos ^2\vartheta ), \end{aligned}$$

and the non-diagonal elements of the coefficient matrix of the collective decay can be approximated to be $\gamma$, i.e. $\gamma _{12}=$ $\gamma _{21}$ $\approx$ $\gamma$. Below we provide^2^ the optical frequency $\omega \approx 3 \times 10^{15}$ Hz and dipole moment $\approx 1$ *Debye*. In Fig. 2, we provide $D/\lambda$ $=0.01$, $N=20$, $n=400$, $g/\lambda$ $=0$ (blue), $g/\lambda$ $=1$ (pink), $g/\lambda$ $=2.5$ (brown), $g/\lambda$ $=10$ (red) and the maximal time is $\lambda t_m$ $=2$. (a) $\gamma /\lambda$ $=\frac{1}{4}$; (b) $\gamma /\lambda$ $=\frac{1}{20}$. The estimated phase $\phi$ $=\pi /5$, and its difference interval $\delta \phi$ $=0.0001$. Using Eq. (31), we can obtain dipole-dipole coupling of $g/\gamma =10$ corresponds to interatomic distance $\approx 292$ nm between two atoms, which is the situation of brown line of Fig. 2a. $g/\gamma =40$ (red line of Fig. 2a), 50 (brown line of Fig. 2b) and 200 (red line of Fig. 2b) correspond to the distance $\approx$ 184 nm, $\approx$ 171 nm and $\approx$ 108 nm, respectively.

From Fig. 2, no mater in (a) or (b), we can find that when other parameters are the same and at the same time. If *g* ( i.e. the *d*–*d* interaction intensity of the two atoms) becomes larger, their QFI becomes larger. This can be used in the open quantum systems to protect the QFI, i.e. at the environment of noises of phase diffusion. This is one result of this paper. Besides, comparing (a) with (b), we can find that smaller decay rate $\gamma$ leads to larger QFI, when other circumstances are the same. And this is the other result of this paper.

**Figure 3:**
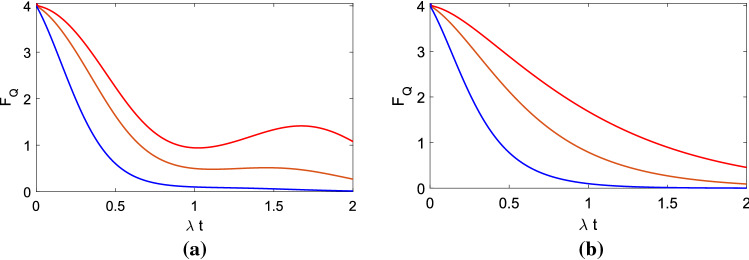
QFI vs. $\lambda t$. Here we provide $g/\lambda$ $=2.5$, $\gamma /\lambda$ $=\frac{1}{20}$ (red), $\gamma /\lambda$ $=\frac{1}{4}$ (brown), $\gamma /\lambda =\frac{3}{4}$ (blue). (**a**) $D/\lambda$ $=0.01$; (**b**) $D/\lambda$ $=5$.

Figure 3 is QFI vs. $\lambda t$. We provide $g/\lambda$ $=2.5$, $N=20$, $n=400$, the estimated phase $\phi =$ $\pi /5$, its difference interval $\delta \phi$ $=0.0001$, and the maximal time is $\lambda t_m$ $=2$. Here $\gamma /\lambda$ $=\frac{1}{20}$ (red); $\gamma /\lambda =\frac{1}{4}$ (brown); $\gamma /\lambda =3/4$ (blue). It is easy to obtain, for example, the blue lines of (a) and (b) correspond to interatomic distance $\approx 195$ nm between two atoms. From Fig. 3a, it is clear that when the decay rate $\gamma$ is larger, under the same parameters, the QFI is smaller. So decreasing the decay rate is helpful to increase the QFI of the system. From our former discussion^1^, we find that when $0\le D/\lambda \le 2.606$, there exists the information backflow to the system, so the QFI has a recovery process from a small value to a big value with time. Here comparing three curves with different $\gamma$ in Fig. 3a, the non-Markovian effect can only emerge when the collective decay rate $\gamma$ is not big, or this effect will be covered up for a big $\gamma$. In Fig. 3b, $D/\lambda$ $=5$, according to the former researches, it belongs to Markovian region of the phase noises. Obviously, it does not exist the information backflow and the QFI recovery anymore. And the curves decays all the time, not like the red curve in Fig. 3a which exists the non-Markovian recovery when the collective decay rate is small.

## Conclusion

In this paper, we have studied the dynamical characters of parament estimation of two atoms driven by PNLs. In the former research^1^ we did not consider *d*–*d* interaction between the two atoms, and we got the analytical expression of QFI. Here we consider this factor and use the numerical simulation in calculating the QFI under PNLs in this paper. For the circumstance of this PNLs driving, we find that the smaller collective decay rate, the larger QFI of two atoms can be, when other parameters are the same. Besides, when other parameters are the same, the larger *d*–*d* interaction of two atoms, the larger QFI of two atoms can be. So we can give a conclusion that collective decay is destructive to QFI of the two atoms, while the *d*–*d* interaction is useful in keeping their QFI. This conclusion may be used in the open quantum system to protect QFI under the PNLs environment. What is more, our scheme gives a general method to solve the model about two atoms with PNLs ultimately, which is suitable for not only two non-interaction atoms but also for two interaction atoms if the Hamiltonian can be written out. And the general numeric solution method of the evolved two-atomic density matrix can be applied to other areas. Knowing the evolved density matrix of the system, many physical problems can be solved, i.e., our general method to calculate the evolved density matrix can be used not only for QFI problems, but also for other physical problems such as fidelity, quantum discord, non-Markovianity and so on.

## Supplementary Information

Supplementary Information.
