# Supplementary material for: Quantum Fisher information of two atoms with dipole–dipole interaction under the environment of phase noise lasers
Source: Sci Rep. 2021 Oct 27;11:21138. doi: 10.1038/s41598-021-99449-9 (PMC8551341; doi:10.1038/s41598-021-99449-9)
Supplement: Supplementary file 1 — Supplementary Information. [file 41598_2021_99449_MOESM1_ESM.docx]

**Supplementary I: definitions about the bases of matrices, and for two atoms**

Here we give our definitions about the bases of matrices , and , e.g.,and $\left(\tilde{L}_{i_{2};i_{1},i_{2};i_{1}}\right)_{i_{2};i_{1},i_{2};i_{1}}$ which are block matrix with block subscripts $i_{2}$ and $i_{1}$. And the definitions are very important for solving the Eq.(21) in the main text. Note that the rearrangement of the matrix ,and $\left(\tilde{L}_{i_{2};i_{1},i_{2};i_{1}}\right)_{i_{2};i_{1},i_{2};i_{1}}$ is according to the basis rearrangement. We provide that $N_{l}$ $(l=1,2)$ is the number of the phase difference intervals of the $l$-th PNL.

For two atoms，or two qubits, the basis of two-qubit density matrix is：

$$\left(E_{i_{1},i_{2}}\right)_{i_{1},i_{2}}\equiv\left(E_{i_{1},i_{2}}\right)_{1\leq i_{1}\leq N_{1},1\leq i_{2}\leq N_{2}}=$$

$$=\left(\begin{matrix} \left(\begin{matrix} e_{00}^{i_{1}} & e_{01}^{i_{1}} \\ e_{10}^{i_{1}} & e_{11}^{i_{1}} \end{matrix}\right)_{i_{1}=1} \\ \vdots\\ \left(\begin{matrix} e_{00}^{i_{1}} & e_{01}^{i_{1}} \\ e_{10}^{i_{1}} & e_{11}^{i_{1}} \end{matrix}\right)_{i_{1}=N_{1}} \end{matrix}\right)\otimes\left(\begin{matrix} \left(\begin{matrix} e_{00}^{i_{2}} & e_{01}^{i_{2}} \\ e_{10}^{i_{2}} & e_{11}^{i_{2}} \end{matrix}\right)_{i_{2}=1} & \cdots& \left(\begin{matrix} e_{00}^{i_{2}} & e_{01}^{i_{2}} \\ e_{10}^{i_{2}} & e_{11}^{i_{2}} \end{matrix}\right)_{i_{2}=N_{2}} \end{matrix}\right)$$

=$\left(\begin{matrix} \left(\begin{matrix} e_{00}^{i_{1}} & e_{01}^{i_{1}} \\ e_{10}^{i_{1}} & e_{11}^{i_{1}} \end{matrix}\right)_{i_{1}=1}\otimes\left(\begin{matrix} e_{00}^{i_{2}} & e_{01}^{i_{2}} \\ e_{10}^{i_{2}} & e_{11}^{i_{2}} \end{matrix}\right)_{i_{2}=1} & \cdots& \left(\begin{matrix} e_{00}^{i_{1}} & e_{01}^{i_{1}} \\ e_{10}^{i_{1}} & e_{11}^{i_{1}} \end{matrix}\right)_{i_{1}=1}\otimes\left(\begin{matrix} e_{00}^{i_{2}} & e_{01}^{i_{2}} \\ e_{10}^{i_{2}} & e_{11}^{i_{2}} \end{matrix}\right)_{i_{2}=N_{2}} \\ \vdots& \ddots& \vdots\\ \left(\begin{matrix} e_{00}^{i_{1}} & e_{01}^{i_{1}} \\ e_{10}^{i_{1}} & e_{11}^{i_{1}} \end{matrix}\right)_{i_{1}=N_{1}}\otimes\left(\begin{matrix} e_{00}^{i_{2}} & e_{01}^{i_{2}} \\ e_{10}^{i_{2}} & e_{11}^{i_{2}} \end{matrix}\right)_{i_{2}=1} & \cdots& \left(\begin{matrix} e_{00}^{i_{1}} & e_{01}^{i_{1}} \\ e_{10}^{i_{1}} & e_{11}^{i_{1}} \end{matrix}\right)_{i_{1}=N_{1}}\otimes\left(\begin{matrix} e_{00}^{i_{2}} & e_{01}^{i_{2}} \\ e_{10}^{i_{2}} & e_{11}^{i_{2}} \end{matrix}\right)_{i_{2}=N_{2}} \end{matrix}\right)$

$$=\left(\begin{matrix} \left(\begin{matrix} e_{00}^{i_{1}}\left(\begin{matrix} e_{00}^{i_{2}} & e_{01}^{i_{2}} \\ e_{10}^{i_{2}} & e_{11}^{i_{2}} \end{matrix}\right)_{i_{2}=1} & e_{01}^{i_{1}}\left(\begin{matrix} e_{00}^{i_{2}} & e_{01}^{i_{2}} \\ e_{10}^{i_{2}} & e_{11}^{i_{2}} \end{matrix}\right)_{i_{2}=1} \\ e_{10}^{i_{1}}\left(\begin{matrix} e_{00}^{i_{2}} & e_{01}^{i_{2}} \\ e_{10}^{i_{2}} & e_{11}^{i_{2}} \end{matrix}\right)_{i_{2}=1} & e_{11}^{i_{1}}\left(\begin{matrix} e_{00}^{i_{2}} & e_{01}^{i_{2}} \\ e_{10}^{i_{2}} & e_{11}^{i_{2}} \end{matrix}\right)_{i_{2}=1} \end{matrix}\right)_{i_{1}=1} & \cdots& \left(\begin{matrix} e_{00}^{i_{1}}\left(\begin{matrix} e_{00}^{i_{2}} & e_{01}^{i_{2}} \\ e_{10}^{i_{2}} & e_{11}^{i_{2}} \end{matrix}\right)_{i_{2}=N_{2}} & e_{01}^{i_{1}}\left(\begin{matrix} e_{00}^{i_{2}} & e_{01}^{i_{2}} \\ e_{10}^{i_{2}} & e_{11}^{i_{2}} \end{matrix}\right)_{i_{2}=N_{2}} \\ e_{10}^{i_{1}}\left(\begin{matrix} e_{00}^{i_{2}} & e_{01}^{i_{2}} \\ e_{10}^{i_{2}} & e_{11}^{i_{2}} \end{matrix}\right)_{i_{2}=N_{2}} & e_{11}^{i_{1}}\left(\begin{matrix} e_{00}^{i_{2}} & e_{01}^{i_{2}} \\ e_{10}^{i_{2}} & e_{11}^{i_{2}} \end{matrix}\right)_{i_{2}=N_{2}} \end{matrix}\right)_{i_{1}=1} \\ \vdots& \ddots& \vdots\\ \left(\begin{matrix} e_{00}^{i_{1}}\left(\begin{matrix} e_{00}^{i_{2}} & e_{01}^{i_{2}} \\ e_{10}^{i_{2}} & e_{11}^{i_{2}} \end{matrix}\right)_{i_{2}=1} & e_{01}^{i_{1}}\left(\begin{matrix} e_{00}^{i_{2}} & e_{01}^{i_{2}} \\ e_{10}^{i_{2}} & e_{11}^{i_{2}} \end{matrix}\right)_{i_{2}=1} \\ e_{10}^{i_{1}}\left(\begin{matrix} e_{00}^{i_{2}} & e_{01}^{i_{2}} \\ e_{10}^{i_{2}} & e_{11}^{i_{2}} \end{matrix}\right)_{i_{2}=1} & e_{11}^{i_{1}}\left(\begin{matrix} e_{00}^{i_{2}} & e_{01}^{i_{2}} \\ e_{10}^{i_{2}} & e_{11}^{i_{2}} \end{matrix}\right)_{i_{2}=1} \end{matrix}\right)_{i_{1}=N_{1}} & \cdots& \left(\begin{matrix} e_{00}^{i_{1}}\left(\begin{matrix} e_{00}^{i_{2}} & e_{01}^{i_{2}} \\ e_{10}^{i_{2}} & e_{11}^{i_{2}} \end{matrix}\right)_{i_{2}=N_{2}} & e_{01}^{i_{1}}\left(\begin{matrix} e_{00}^{i_{2}} & e_{01}^{i_{2}} \\ e_{10}^{i_{2}} & e_{11}^{i_{2}} \end{matrix}\right)_{i_{2}=N_{2}} \\ e_{10}^{i_{1}}\left(\begin{matrix} e_{00}^{i_{2}} & e_{01}^{i_{2}} \\ e_{10}^{i_{2}} & e_{11}^{i_{2}} \end{matrix}\right)_{i_{2}=N_{2}} & e_{11}^{i_{1}}\left(\begin{matrix} e_{00}^{i_{2}} & e_{01}^{i_{2}} \\ e_{10}^{i_{2}} & e_{11}^{i_{2}} \end{matrix}\right)_{i_{2}=N_{2}} \end{matrix}\right)_{i_{1}=N_{1}} \end{matrix}\right)$$

$=\left(\begin{matrix} E_{1,1} & E_{1,2} & \cdots& E_{1,N_{2}} \\ \vdots& \vdots& \vdots& \vdots\\ E_{N_{1},1} & E_{N_{1},2} & \cdots& E_{N_{1},N_{2}} \end{matrix}\right)$. Above we have used an important definition that odd qubit (i.e., the first qubit) spans its subscripts by row and even qubit (i.e., the second qubit) spans its subscripts by column.

The first rearrangement: we rearrange the blocks of $\left(E_{i_{1},i_{2}}\right)_{i_{1},i_{2}}$ column block by column block into a new column block matrix $\left(E_{i_{2};i_{1},1}\right)_{i_{2};i_{1},1}$ . After the first rearrangement, the basis will be

$\left(E_{i_{2};i_{1},1}\right)_{i_{2};i_{1},1}\equiv\left(E_{i_{2};i_{1},1}\right)_{1\leq i_{2}\leq N_{2};1\leq i_{1}\leq N_{1},1}=$ $\left(\begin{matrix} E_{1;1,1} \\ \vdots\\ E_{1;N_{1},1} \\ E_{2;1,1} \\ \vdots\\ E_{2;N_{1},1} \\ \vdots\\ \vdots\\ E_{N_{2};1,1} \\ \vdots\\ E_{N_{2};N_{1},1} \end{matrix}\right)$ $=\left(\begin{matrix} E_{1;1,1}, & \cdots, & E_{1;N_{1},1}, & E_{2;1,1}, & \cdots, & E_{2;N_{1},1}, & \cdots, & \cdots, & E_{N_{2};1,1}, & \cdots, & E_{N_{2};N_{1},1} \end{matrix}\right)^{T}$. Here $T$ means transposition of matrix in this paper. $\left(E_{i_{2};i_{1},1}\right)_{i_{2};i_{1},1}$is the rearrangement of $\left(E_{i_{1},i_{2}}\right)_{i_{1},i_{2}}$and each of their corresponding blocks is the same, i.e., $E_{i_{2};i_{1},1}$=$E_{i_{1};i_{2}}$=$\left(\begin{matrix} \begin{matrix} e_{00}^{i_{1}}e_{00}^{i_{2}} & e_{00}^{i_{1}}e_{01}^{i_{2}} \\ e_{00}^{i_{1}}e_{10}^{i_{2}} & e_{00}^{i_{1}}e_{11}^{i_{2}} \end{matrix} & \begin{matrix} e_{01}^{i_{1}}e_{00}^{i_{2}} & e_{01}^{i_{1}}e_{01}^{i_{2}} \\ e_{01}^{i_{1}}e_{10}^{i_{2}} & e_{01}^{i_{1}}e_{11}^{i_{2}} \end{matrix} \\ \begin{matrix} e_{10}^{i_{1}}e_{00}^{i_{2}} & e_{10}^{i_{1}}e_{01}^{i_{2}} \\ e_{10}^{i_{1}}e_{10}^{i_{2}} & e_{10}^{i_{1}}e_{11}^{i_{2}} \end{matrix} & \begin{matrix} e_{11}^{i_{1}}e_{00}^{i_{2}} & e_{11}^{i_{1}}e_{01}^{i_{2}} \\ e_{11}^{i_{1}}e_{10}^{i_{2}} & e_{11}^{i_{1}}e_{11}^{i_{2}} \end{matrix} \end{matrix}\right)$. The second rearrangement is to rearrange these elements of each block$E_{i_{2};i_{1},1}$, row element by row element (column element by column element is also permissible), into a column vector $\tilde{E}_{i_{2};i_{1},1}$. So after the second rearrangement, $\left(E_{i_{2};i_{1},1}\right)_{i_{2};i_{1},1}$can be changed into $\left(\tilde{E}_{i_{2};i_{1},1}\right)_{i_{2};i_{1},1}$ and $\tilde{E}_{i_{2};i_{1},1}=$ $\left(\begin{matrix} e_{00}^{i_{1}}e_{00}^{i_{2}}, & e_{00}^{i_{1}}e_{01}^{i_{2}}, & e_{01}^{i_{1}}e_{00}^{i_{2}}, & e_{01}^{i_{1}}e_{01}^{i_{2}}, & e_{00}^{i_{1}}e_{10}^{i_{2}}, & e_{00}^{i_{1}}e_{11}^{i_{2}}, & e_{01}^{i_{1}}e_{10}^{i_{2}}, & e_{01}^{i_{1}}e_{11}^{i_{2}}, & e_{10}^{i_{1}}e_{00}^{i_{2}}, & e_{10}^{i_{1}}e_{01}^{i_{2}}, & e_{11}^{i_{1}}e_{00}^{i_{2}}, & e_{11}^{i_{1}}e_{01}^{i_{2}}, & e_{10}^{i_{1}}e_{10}^{i_{2}}, & e_{10}^{i_{1}}e_{11}^{i_{2}}, & e_{11}^{i_{1}}e_{10}^{i_{2}}, & e_{11}^{i_{1}}e_{11}^{i_{2}} \end{matrix}\right)^{T}$. And

$\left(\tilde{E}_{i_{2};i_{1},1}\right)_{i_{2};i_{1},1}\equiv\left(\tilde{E}_{i_{2};i_{1},1}\right)_{1\leq i_{2}\leq N_{2};1\leq i_{1}\leq N_{1},1}=$ $\left(\begin{matrix} \tilde{E}_{1;1,1} \\ \vdots\\ \tilde{E}_{1;N_{1},1} \\ \tilde{E}_{2;1,1} \\ \vdots\\ \tilde{E}_{2;N_{1},1} \\ \vdots\\ \vdots\\ \tilde{E}_{N_{2};1,1} \\ \vdots\\ \tilde{E}_{N_{2};N_{1},1} \end{matrix}\right)$ $=\left(\begin{matrix} \tilde{E}_{1;1,1}, & \cdots, & \tilde{E}_{1;N_{1},1}, & \tilde{E}_{2;1,1}, & \cdots, & \tilde{E}_{2;N_{1},1}, & \cdots, & \cdots, & \tilde{E}_{N_{2};1,1}, & \cdots, & \tilde{E}_{N_{2};N_{1},1} \end{matrix}\right)^{T}$, which is the basis of $=(\tilde{\rho}^{n}_{i_{2};i_{1},1})_{i_{2};i_{1},1}$ or the basis of $=(\tilde{\rho}^{n-1}_{i_{2};i_{1},1})_{i_{2};i_{1},1}$.

For $N_{1}N_{2}$ noise combinations of two PNLs initially, i.e. the combinations of possible values for $1\leq i_{2}\leq N_{2}$ and $1\leq i_{1}\leq N_{1}$, the initial density matrices of two qubits are blocks of$=\left(\begin{matrix} \tilde{\rho}^{0}_{1;1,1}, & \cdots, & \tilde{\rho}^{0}_{1;N_{1},1}, & \tilde{\rho}^{0}_{2;1,1}, & \cdots, & \tilde{\rho}^{0}_{2;N_{1},1}, & \cdots, & \cdots, & \tilde{\rho}^{0}_{N_{2};1,1}, & \cdots, & \tilde{\rho}^{0}_{N_{2};N_{1},1} \end{matrix}\right)^{T}$, and each block is the same. This means the initial matrices of two quibits for different phase noise combinations are the same. For example, we can provide $\tilde{\rho}^{0}_{i_{2};i_{1},1}$, for any values of $1\leq i_{2}\leq N_{2}$ and $1\leq i_{1}\leq N_{1}$.

The block indexes of mean the block rearranging sequence of the blocks of by the sequence value of $(i_{2}-1)*N_{1}$ $+(i_{1}-1)$ from $0$ to $N_{1}N_{2}-1$. is the diagonal block matrix where the diagonal blocks follow the same sequence of , i.e., by the same sequence value of $(i_{2}-1)*N_{1}$ $+(i_{1}-1)$ from $0$ to $N_{1}N_{2}-1$. For example, the basis of $\left(\tilde{L}_{i_{2};i_{1},i_{2};i_{1}}\right)_{i_{2};i_{1},i_{2};i_{1}}$is $\left(\tilde{E}_{i_{2};i_{1},i_{2};i_{1}}\right)_{i_{2};i_{1},i_{2};i_{1}}$which is

$\left(\tilde{E}_{i_{2};i_{1},i_{2};i_{1}}\right)_{i_{2};i_{1},i_{2};i_{1}}\equiv\left(\tilde{E}_{i_{2};i_{1},i_{2};i_{1}}\right)_{1\leq i_{2}\leq N_{2};1\leq i_{1}\leq N_{1},1\leq i_{2}\leq N_{2};1\leq i_{1}\leq N_{1}}=$ $\left(\begin{matrix} \tilde{E}_{1;1,1;1} & & & & & & & & & & \\ & \ddots& & & & & & & & & \\ & & \tilde{E}_{1;N_{1},1;N_{1}} & & & & & & & & \\ & & & \tilde{E}_{2;1,2;1} & & & & & & & \\ & & & & \ddots& & & & & & \\ & & & & & \tilde{E}_{2;N_{1},2;N_{1}} & & & & & \\ & & & & & & \ddots& & & & \\ & & & & & & & \ddots& & & \\ & & & & & & & & \tilde{E}_{N_{2};1,N_{2};1} & & \\ & & & & & & & & & \ddots& \\ & & & & & & & & & & \tilde{E}_{N_{2};N_{1},N_{2};N_{1}} \end{matrix}\right)$.

From the above we know that the first rearrangement is rearranging the positions of blocks of block matrix into . And the second rearrangement is rearranging the positions of the elements inside each block of , e.g. changing $E_{i_{2};i_{1},1}$ into $\tilde{E}_{i_{2};i_{1},1}$ and changing $L_{i_{2};i_{1},1}$ $\rho_{i_{2};i_{1},1}^{n}$ into$\tilde{L}_{i_{2};i_{1},i_{2};i_{1}}\tilde{\rho}_{i_{2};i_{1},1}^{n}$ ( thus we can separate $L_{i_{2};i_{1},1}$ $\rho_{i_{2};i_{1},1}^{n}$ into block $\tilde{L}_{i_{2};i_{1},i_{2};i_{1}}$ and block $\tilde{\rho}_{i_{2};i_{1},1}^{n}$ multiplying ).
